# Alpha-1 antitrypsin deficiency and pregnancy complications and birth outcomes: A population-based cohort study in Denmark

**DOI:** 10.1371/journal.pone.0296434

**Published:** 2024-01-02

**Authors:** Helen T. Orimoloye, Di He, Tong Li, Carla Janzen, Igor Barjaktarevic, Xuexia Wang, Johnni Hansen, Julia E. Heck

**Affiliations:** 1 Department of Rehabilitation and Health Services, University of North Texas, Denton, Texas, United States of America; 2 Department of Epidemiology, Fielding School of Public Health, University of California, Los Angeles, Los Angeles, California, United States of America; 3 Department of Obstetrics and Gynecology, University of California, Los Angeles, Los Angeles, California, United States of America; 4 Division of Pulmonary and Critical Care, University of California, Los Angeles, Los Angeles, California, United States of America; 5 Department of Biostatistics, Florida International University, Miami, Florida, United States of America; 6 Danish Cancer Society, Copenhagen, Denmark; Universita Politecnica delle Marche, ITALY

## Abstract

**Background:**

Alpha-1 antitrypsin deficiency (AATD) is related to developing lung and liver disease, but no large-scale studies examine its association with birth outcomes.

**Objective:**

We investigated the risk of pregnancy complications and adverse birth outcomes in mothers and children with AATD.

**Methods:**

Using a large cohort data of Danish mothers and children with AATD from 1973 to 2013 (n = 2,027,229), with 559 cases (305 mothers and 254 children). We conducted Poisson regression to examine associations between alpha-1 antitrypsin deficiency, adverse birth outcomes, and pregnancy complications in mothers and children.

**Results:**

AATD was related to term low birth weight [<2500g; Risk Ratio(RR) = 2.04, 95% confidence interval (CI): 1.50–2.79], lowest quartile of abdominal circumference at birth in children of non-smoking mothers (RR = 1.55, 95% CI: 1.14–2.11), delivery via Cesarean-section (RR = 1.59, 95% CI: 1.05–2.40), preterm birth (RR = 1.54, 95% CI: 1.19–2.00) and preeclampsia (RR = 2.64, 95% CI: 1.76–3.94).

**Conclusions:**

This emphasizes the need for mothers with AATD to be monitored closely during pregnancy to reduce the risk of adverse birth outcomes. Routine screening for alpha-1 antitrypsin in pregnancy may be considered among mothers with a pulmonary and liver disease history.

## Introduction

Alpha-1 antitrypsin deficiency (AATD) is an autosomal codominant inherited condition characterized by low blood alpha-1 antitrypsin (AAT) levels. It is among the three most common potentially lethal genetic diseases globally. The condition affects 189 million carriers and 1.45 million persons globally [1]. The underlying pathology is the presence of one or both mutations in the alleles coding for AAT, which leads to dysfunctional protein function and hepatic accumulation of polymerized abnormal protein [2].

AATD affects the lungs and liver and can cause pan-acinar emphysema, premature-onset chronic obstructive pulmonary disease (COPD), and liver cirrhosis with significant heterogeneity in disease presentation. A few studies have examined pregnancy complications and birth outcomes among mothers with AATD [3, 4], reporting that either AATD or lower levels of AAT in the serum or amniotic fluid of pregnant mothers are associated with pregnancy complications and adverse birth outcomes in the child. Adverse impacts in mothers included a deterioration in maternal respiratory function, spontaneous pneumothorax, miscarriage, and pre-eclampsia [4]. In offspring, intrauterine growth restriction (IUGR) and increased nuchal translucency were reported [5, 6]. Prior studies have reported adverse birth outcomes due to asthma [7]. However, none have considered AATD as a factor. There have been no large-scale studies on pregnancy and birth-related complications in people with AATD. This study investigated the epidemiology of AATD-related pregnancies, focusing on pregnancy complications and birth outcomes in a large cohort study of 2 million Danish children.

## Methods

This cohort study included subjects taken from several occupational epidemiologic studies in Denmark. Participants (n = 2,027,229 children and their parents) were taken from Danish national registries from births from 1973–2013. The children’s biological mothers were identified from the Medical Birth Register, and demographic information was taken from the Central Person Register. AATD diagnoses (270.80, E88, E88.0, E88.0A, E88.0B) and congenital anomalies (ICD codes 74099–75999, DQ850, DQ265, DQ660, DQ838, DQ442, DQ658F, DQ531, DQ411, DQ701) were ascertained using the modified Danish version of the tenth revision of the International Classification of Diseases (ICD-8 and ICD-10 coding) in the Danish National Patient Register.

**Fig 1:**
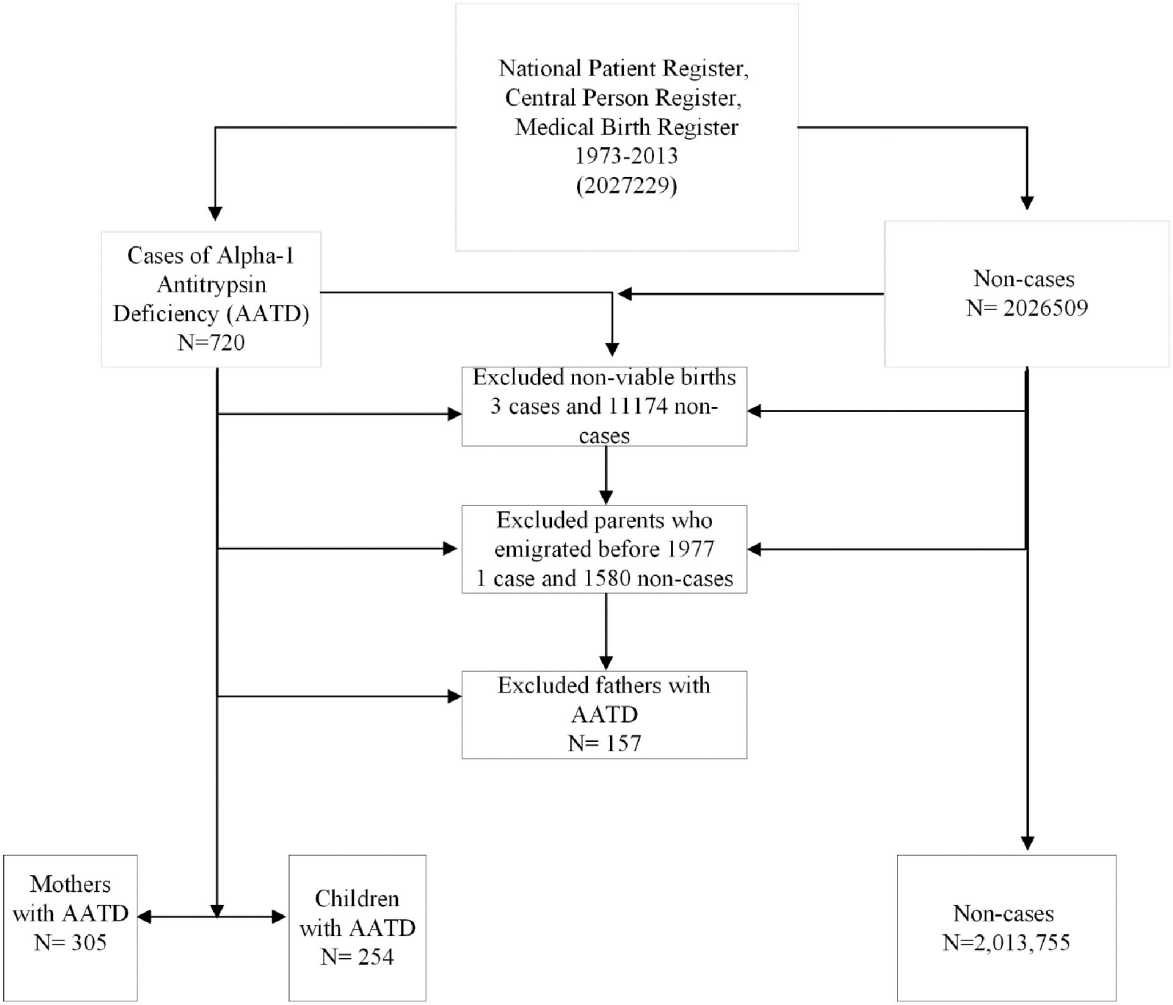
Flow chart of AATD cases and non-cases. This Figure shows the selection of cases and non-cases from Danish registries and the inclusion and exclusion criteria.

We did not have details on the genotype of AATD, and no one in our sample had a record of using augmentation therapy (Danish procedure code = ZZ1980). The registries were linked via the 10-digit unique personal identification number assigned by the Central Population Registry. We excluded children with non-viable birth weights (<500 g) or gestational ages between <21 or >46 weeks (3 cases and 11174 non-cases). We excluded 1 case and 1580 non-cases whose parents died or emigrated before 1977, before the establishment of the National Patient Register, and we also excluded families of fathers with AATD (n = 157). Thus, our final sample included 559 cases (305 mothers and 254 children) and 2013755 non-cases (Fig 1).

This study was approved by the institutional review board of the University of North Texas and the University of California Los Angeles and a waiver of informed consent was obtained. Further, this study follows the legislation from the Danish Data Protection Agency.

Our outcomes of interest were the placenta/birth weight ratio, which indicates maternal and neonatal morbidity, and the placenta’s ability to maintain an adequate nutrient supply to the fetus; preeclampsia–characterized by elevated blood pressure and protein excretion in the urine; preterm rupture of membranes (PROM)–defined as the rupture of membranes before the onset of labor. Other pregnancy complications in mothers and potential confounding variables, such as maternal pregnancy history, tobacco smoking, birth weight, gestational age, and other gestational factors, were ascertained from the Medical Births Registry (1973+). These conditions were identified using ICD-8 and ICD-10 codes, shown in S1 Table in S1 File.

Fetal growth metrics such as birth weight, placenta weight, ponderal index, birth length, and abdominal circumference were put into quartiles or grouped based on the distribution in non-cases; due to rounding to the nearest cm or 10g in the Medical Births Registry for some variables, the percent of non-cases in each quartile was not equivalent to 25% in all instances. Analyses began with descriptive statistics of variables of interest and potential confounders. Next, the prevalence (N%) and risk ratios with 95% confidence intervals for pregnancy complications and adverse birth outcomes with AATD were estimated using Poisson regression in crude and adjusted models.

The selection of covariates and potential confounders was based on the literature and the examination of data [8]. We included covariates in models that changed beta values by 10% or more [9]. Final models adjusted for maternal age (continuous), birth year (1973–1982, 1983–1992, 1993–2002, >2002), and maternal smoking status (smoking at the first prenatal visit, Yes or No). We considered adjustment for other covariates, alpha-1-related conditions [10], including chronic bronchitis, hepatic cirrhosis, alcoholic cirrhosis, all-cause cirrhosis, prolonged oxygen dependency, dyspnea, emphysema, all-cause fatty liver disease, atopic dermatitis, rhinitis, panniculitis, pneumothorax, and vasculitis [11, 12]. However, adjustments for these factors did not change point estimates by more than 10% and were not included in the final models. We then separately examined the risk of these alpha-1-related conditions in our study subjects.

Due to changes in Danish tax law, family socioeconomic status obtained from the Central Person Register had large and increasing missing values across the study period (1973–1982: 87.1% missing; 1983–1992: 92.1%; 1993–2002: 99.6%; 2002+: 100%) and was not included in models. All analyses were conducted using SAS 9.4 software.

Smoking increases the likelihood of AATD symptoms and AATD diagnosis [13]. Likewise, consistent associations have been reported between restricted fetal growth and maternal smoking during pregnancy. Therefore, we stratified by maternal smoking status to determine whether this subgroup’s results differed. Maternal smoking status was available for births between 1991–2013 only. Therefore, we estimated smoking status when it was missing by multiple imputation using a model that included gestational age, child’s birth length, ponderal index, placental weight, child’s abdominal circumference, child’s birth weight, and birth year as the predictors. In a validation analysis of the imputed status, we compared the results adjusting for smoking with and without imputation in sensitivity analyses.

We did a sensitivity analysis for children born at term to mothers with AATD, mothers without pre-eclampsia, or PROM to determine whether IUGR was present in these groups. To determine whether conditions associated with AATD might have worsened during pregnancy, we examined these conditions using ICD codes from the Danish National Patient Register (S1 Table in S1 File). Finally, in an exploratory analysis, we determined if congenital anomalies were more common among infants with AATD.

## Results

Table 1 shows the demographic characteristics of the study participants. The average maternal age at index childbirth was 28.2 years [standard deviation (SD) = 5.1] years, and mothers were diagnosed at an average of 16.0 (SD = 12.3) years after the index child was born. Of mothers with AATD, 29 (9.5%) were diagnosed before the index child’s birth. Likely because AATD is often diagnosed in midlife or later [14], parental cases of AATD were more common among those born earlier in the study period. Compared to non-cases, cases were more likely to have been born in Denmark and to reside in small towns or rural areas.

**Table 1:** Demographic characteristics of Alpha-1-antitrypsin deficiency (AATD) cases and non-cases.

	All subjects				Non-smoking mothers only ^1^			
	Non-cases	AATD Cases			Non-cases	AATD Cases		
		Mother	Children	All ^†^		Mother	Children	All ^†^
	N = 2013755	N = 305	N = 254	N = 559	N = 833900	N = 78	N = 65	N = 143
Child’s Sex								
Male	1036608 (51.5)	162 (53.1)	137 (53.9)	299 (53.5)	428285 (51.4)	39 (50.0)	30 (46.2)	69 (48.3)
Female	977147 (48.5)	143 (46.9)	117 (46.1)	260 (46.5)	405615 (48.6)	39 (50.0)	35 (53.9)	74 (51.7)
Birth year								
1973–1982	505225 (25.1)	115 (37.7)	108 (42.5)	223 (39.9)	---	---	---	---
1983–1992	479350 (23.8)	86 (28.2)	65 (25.6)	151 (27.0)	63874 (7.7)	6 (7.7)	5 (7.7)	11(7.7)
1993–2002	540229 (26.8)	59 (19.3)	45 (17.7)	104 (18.6)	374731 (44.9)	34 (43.6)	27 (41.5)	61 (42.7)
>2002	488951 (24.3)	45 (14.8)	36 (14.2)	81 (14.5)	395295 (47.4)	38 (48.7)	33 (50.8)	71 (49.6)
Urban/Rural^2^								
Urban	644189 (32.0)	96 (31.5)	72 (28.4)	168 (30.05)	289724 (34.7)	28 (35.9)	16 (24.6)	44 (30.8)
Small towns	634669 (31.5)	96 (31.5)	98 (38.6)	194 (34.7)	231682 (27.8)	23 (29.5)	23 (35.4)	46 (32.1)
Rural	734897 (36.5)	113 (37.1)	84 (33.1)	197 (35.2)	312494 (37.5)	27 (34.6)	26 (40.0)	53 (37.1)
Maternal age								
24 or less	421408 (20.9)	81 (26.6)	77 (30.3)	158 (28.3)	81008 (9.7)	12 (15.4)	7 (10.8)	19 (13.3)
25–29	734987 (36.5)	95 (31.2)	84 (33.1)	179 (32.0)	282891 (33.9)	23 (29.5)	25 (38.5)	48 (33.5)
30–34	592710 (29.4)	87 (28.5)	74 (29.1)	161 (28.8)	315630 (37.9)	30 (38.5)	26 (40.0)	56 (39.2)
>34	264650 (13.1)	42 (13.8)	19 (7.5)	61 (10.9)	154371 (18.5)	13 (16.7)	7 (10.8)	20 (14.0)
Paternal age								
24 or less	197992 (9.9)	38 (12.5)	35 (13.9)	73 (13.1)	35227 (4.3)	<5 (5.2)	6 (9.4)	10 (7.1)
25–29	595098 (29.8)	101 (33.2)	86 (34.1)	187 (33.6)	193706 (23.4)	16 (20.8)	19 (29.7)	35 (24.8)
30–34	672069 (33.6)	92 (30.3)	85 (33.7)	177 (31.8)	316397 (38.2)	32 (41.6)	24 (37.5)	56 (39.7)
>34	533214 (26.7)	73 (24.0)	46 (18.3)	119 (21.4)	282106 (34.1)	25 (32.5)	15 (23.4)	40 (28.4)
Mother’s birthplace								
Denmark	1865001 (92.8)	293 (96.4)	247 (97.2)	540 (96.1)	754430 (90.6)	74 (94.9)	62 (95.4)	221 (93.3)
Other	144616 (7.2)	11 (3.6)	7 (2.8)	18 (4.0)	77920 (9.4)	<5	<5	16 (6.8)
Father’s birthplace								
Denmark	1846972 (92.7)	286 (94.4)	245 (97.6)	879 (96.7)	748395 (90.7)	75 (97.4)	61 (95.3)	227 (96.6)
Other	146095 (7.3)	17 (5.6)	6 (2.4)	30 (3.3)	77070 (9.3)	<5	<5	8 (3.4)
Family SES^2^								
High	195363 (13.7)	35 (15.0)	18 (8.6)	88 (12.1)	75466 (17.2)	6 (17.1)	<5	18 (14.5)
Medium-high	247092 (17.4)	38 (16.2)	36 (17.1)	120 (16.5)	91109 (20.8)	8 (22.9)	<5	22 (17.7)
Medium	266274 (18.7)	52 (22.2)	38 (18.1)	151 (20.7)	77636 (17.7)	5 (14.3)	6 (19.4)	23 (18.6)
Medium-low	434688 (30.6)	77 (32.9)	76 (36.2)	245 (33.7)	111899 (25.5)	11 (31.4)	8 (25.8)	34 (27.4)
Low	278777 (19.6)	32 (13.7)	42 (20.0)	124 (17.0)	81963 (18.7)	5 (14.3)	11 (35.5)	27 (21.8)

^**†**^ Families with mother cases or/and children cases

^1^ Defined as no smoking at the first prenatal visit. Collected for births 1991+;

^2^ Family SES: due to changes in Danish tax law, this variable had increasing missing across the study period (1973–1982: 87.1% missing; 1983–1992: 92.1%; 1993–2002: 99.6%; 2002+: 100%). This variable is not presented among nonsmokers because the periods that the two variables were collected did not overlap. High: Academics and high-level self-employed; Medium-high: Middle-long education; Medium: Shorter education; Medium-low: Skilled worker; Low: Unskilled worker.

Less than 1% were missing

### Maternal AATD and risk for adverse birth outcomes

Table 2 shows the maternal and child health characteristics of the cases and non-cases, while the crude and multivariable analyses of risk for adverse birth outcomes are shown in Table 3. Offspring of mothers with AATD had an increased risk of low birth weight and a higher placenta/birth weight ratio, shorter birth length, and smallest quartile of the ponderal index. There was a higher risk of decreased abdominal circumference at birth in babies from mothers with AATD who did not smoke. There was also a higher risk of delivery via Cesarean section and preterm birth among mothers with AATD.

**Table 2:** Percentage distribution of maternal and child health of AATD * cases and non-cases.

	Non-cases	Cases	
	N = 2013755	Mother (N = 305)	Children (N = 254)
***Maternal Health***			
**Number of previous live births**			
**0**	1380067 (68.5)	221 (72.5)	173 (68.1)
**1**	484300 (24.1)	62 (20.3)	64 (25.2)
**>1**	149388 (7.4)	22 (7.2)	17 (6.7)
**Maternal smoking at the first prenatal visit** ^1^	238443 (22.2)	33 (29.7)	24 (27.0)
**Maternal history of miscarriage**	290281 (14.4)	37 (12.1)	35 (13.8)
**Mother’s height (cm)** ^2^ Mean (SD)	168.3 (6.4)	165.8 (7.2)	
**Mother’s weight (kg)** ^2^ Mean (SD)	68.9 (14.9)	67.9 (15.7)	
**Maternal BMI** ^2^ Mean (SD)	24.3 (5.0)	24.7 (5.0)	
**Maternal Overweight (BMI≥25)** ^2^ (Yes)	136249 (33.0)	14 (36.8)	
***Child Health***			
**Birthweight (g)** Mean (SD)	3405.3 (602.1)	3146.3 (664.4)	3193.0 (620.7)
<2500	103337 (5.1)	35 (11.5)	23 (9.1)
2500–4000	1645213 (81.7)	250 (82.0)	210 (82.7)
>4000	265205 (13.2)	20 (6.6)	21 (8.3)
**Placenta weight (g)** Mean (SD)	683.2 (170.8)	686.8 (137.7)	705.9 (180.1)
<570	189826 (24.1)	15 (18.8)	13 (23.2)
570–650	197281 (25.1)	18 (22.5)	10 (17.9)
660–760	199606 (25.4)	29 (36.3)	16 (28.6)
>760	200438 (25.5)	18 (22.5)	17 (30.4)
**Placental weight/birth weight ratio** Mean (SD)	0.2 (0.1)	0.2 (0.1)	0.2 (0.1)
1st quartile	196615 (25.0)	18 (22.5)	11 (19.6)
2nd quartile	197269 (25.1)	17 (21.3)	14 (25.0)
3rd quartile	196495 (25.0)	14 (17.5)	12 (21.4)
4th quartile	196772 (25.0)	31 (38.8)	19 (33.9)
**Birth length (cm)** Mean (SD)	51.7 (2.7)	50.9 (3.1)	51.2 (2.8)
<51	567501 (28.4)	115 (37.8)	87 (34.8)
51	296448 (14.9)	51 (16.8)	49 (19.6)
52–53	665413 (33.3)	86 (28.3)	71 (28.4)
>53	467027 (23.4)	52 (17.1)	43 (17.2)
**Head circumference at birth** Mean (SD)	35.0 (1.9)	34.9 (2.0)	35.1 (1.8)
<34	130887 (16.5)	14 (17.7)	7 (13.0)
34–35	343731 (43.4)	35 (44.3)	27 (50.0)
36	172226 (21.7)	16 (20.3)	9 (16.7)
>36	145958 (18.4)	14 (17.7)	11 (20.4)
**Abdominal circumference at birth (cm)** Mean (SD)	33.2 (2.4)	32.7 (2.5)	33.1 (2.2)
<32	156449 (20.4)	19 (25.0)	13 (24.5)
32–33	246259 (32.1)	29 (38.2)	17 (32.1)
34	144562 (18.8)	10 (13.2)	12 (22.6)
>34	220715 (28.7)	18 (23.7)	11 (20.8)
**Ponderal Index** ^3^ Mean (SD)	2.5 (0.4)	2.4 (0.3)	2.4 (0.3)
1st quartile	499003 (25.0)	123 (40.5)	97 (38.8)
2nd quartile	499074 (25.0)	71 (23.4)	61 (24.4)
3rd quartile	499392 (25.0)	61 (20.1)	48 (19.2)
4th quartile	498920 (25.0)	49 (16.1)	44 (17.6)
**Cesarean section**	72963 (7.9)	19 (12.0)	7 (5.3)
**Gestational age (weeks)** Mean (SD)	39.1 (2.2)	38.7 (2.4)	38.8 (2.5)
Preterm birth (≤37)	201870 (10.0)	48 (15.7)	31 (12.2)
**Size for gestational age** ^4^ Mean (SD)	39.1 (2.2)	38.7 (2.4)	38.8 (2.5)
<10%	122356 (10.2)	25 (14.3)	19 (14.8)
10–90%	954430 (79.6)	141 (80.6)	97 (75.8)
≥90%	121851 (10.2)	9 (5.1)	12 (9.4)
**Apgar score, 1 minute** ^5^	9.3 (1.4)	9.1 (1.9)	9.3 (1.4)
<9	127266 (14.9)	25 (17.2)	22 (17.6)

* Alpha-1-antitrypsin deficiency;

^1^ Collected for 1991–2013; Maternal smoking Risk ratio: crude 1.48 (0.99–2.22) adjusted: 1.57 (0.96–2.54)

^2^ Collected for births 2003+;

^3^ Ponderal Index = birth weight (g)/ birth length (cm)³;

^4^ Collected for 1981–2004;

^5^ Collected for 1991 +

Less than 1% were missing

**Table 3:** Multivariable analysis of maternal AATD* and risk for adverse birth outcomes.

	Mother with AATD (all) (N = 305)		Non-smoking mother with AATD (N = 78)	
	RR (95% CI)		RR (95% CI)	
	Crude	Adjusted ‡	Crude	Adjusted ^†^
***Maternal Health***				
**Number of Previous Live Births**				
0	Referent	Referent	Referent	Referent
1	0.84 (0.68–1.05)	0.89 (0.71–1.11)	1.22 (0.89–1.68)	1.16 (0.84–1.60)
>1	0.93 (0.62–1.38)	0.91 (0.63–1.33)	0.72 (0.28–1.84)	0.62 (0.25–1.55)
**Maternal history of miscarriage**:	0.84 (0.62–1.14)	0.89 (0.66–1.20)	0.90 (0.53–1.51)	0.90 (0.53–1.53)
**Maternal History of Stillbirth**:	2.75 (1.05–7.24)	2.48 (0.94–6.54)	-----	-----
**Mother’s height (cm)** ^1^				
<164	1.06 (0.75–1.48)	1.07 (0.76–1.50)	1.10 (0.76–1.59)	1.11 (0.77–1.61)
164–168	Referent	Referent	Referent	Referent
169–172	0.29 (0.08–1.06)	0.29 (0.08–1.05)	0.37 (0.11–1.30)	0.37 (0.11–1.29)
≥173	0.90 (0.57–1.42)	0.90 (0.57–1.42)	1.03 (0.67–1.57)	1.03 (0.67–1.58)
**Mother’s weight (kg)** ^1^				
<59	0.89 (0.52–1.51)	0.89 (0.53–1.50)	0.87 (0.47–1.61)	0.86 (0.46–1.61)
59–65	Referent	Referent	Referent	Referent
66–74	1.10 (0.76–1.59)	1.10 (0.76–1.59)	1.12 (0.75–1.66)	1.12 (0.75–1.67)
≥75	0.74 (0.39–1.41)	0.75 (0.39–1.45)	0.86 (0.46–1.59)	0.86 (0.46–1.61)
**Overweight (BMI≥25)** ^1^: Yes	1.12 (0.74–1.69)	1.13 (0.75–1.72)	1.16 (0.74–1.82)	1.18 (0.75–1.85)
***Child Health***				
***Birthweight (g)***				
*<2500*	2.08 (1.52–2.83)	2.04 (1.50–2.79)	3.14 (1.83–5.41)	3.11 (1.81–5.35)
2500–4000	Referent	Referent	Referent	Referent
>4000	0.53 (0.35–0.81)	0.60 (0.40–0.91)	0.60 (0.31–1.15)	0.61 (0.32–1.17)
**Birth weight (g) among children born at term**				
<2500	2.43 (1.49–3.96)	2.43 (1.49–3.97)	-----	-----
2500–4000	Referent	Referent	Referent	Referent
>4000	0.56 (0.37–0.85)	0.62 (0.41–0.94)	0.61 (0.32–1.17)	0.62 (0.33–1.19)
**Placenta weight (g)**				
<570	0.93 (0.64–1.35)	0.93 (0.64–1.35)	0.96 (0.65–1.44)	0.96 (0.65–1.44)
570–650	Referent	Referent	Referent	Referent
660–760	1.23 (0.98–1.54)	1.23 (0.98–1.54)	1.17 (0.90–1.53)	1.18 (0.90–1.53)
>760	0.99 (0.72–1.38)	0.99 (0.71–1.37)	0.87 (0.57–1.33)	0.87 (0.57–1.33)
**Placental weight/birth weight ratio (all births)**				
1st quartile	1.12 (0.83–1.53)	1.12 (0.83–1.52)	1.12 (0.80–1.55)	1.12 (0.81–1.56)
2nd quartile	1.09 (0.80–1.51)	1.10 (0.80–1.51)	1.10 (0.78–1.56)	1.11 (0.78–1.57)
3rd quartile	Referent	Referent	Referent	Referent
4th quartile	1.38 (1.13–1.68)	1.37 (1.12–1.67)	1.37 (1.07–1.74)	1.37 (1.07–1.74)
**Birth Length (cm; quartile)**				
<51 (lowest quartile)	1.26 (1.13–1.39)	1.23 (1.11–1.36)	1.41 (1.11–1.79)	1.41 (1.10–1.81)
**Head Circumference at birth (cm; quartile)** ^2^				
<34 (lowest quartile)	1.06 (0.73–1.53)	1.08 (0.74–1.56)	1.10 (0.70–1.72)	1.12 (0.71–1.76)
**Abdominal Circumference at birth (cm; quartile)** ^2^				
<32 (lowest quartile)	1.24 (0.90–1.69)	1.24 (0.89–1.73)	1.54 (1.14–2.10)	1.55 (1.14–2.11)
**Ponderal Index** ^2^^,^^3^				
Lowest quartile	1.18 (1.02–1.37)	1.14 (0.99–1.33)	1.22 (0.91–1.62)	1.21 (0.91–1.61)
**Plural birth**	0.73 (0.24–2.24)	0.81 (0.26–2.49)	0.68 (0.10–4.79)	0.65 (0.09–4.59)
**Cesarean section**	1.53 (1.00–2.33)	1.59 (1.05–2.40)	2.91 (1.72–4.93)	2.46 (1.54–3.91)
**Gestational age (weeks)**				
Preterm birth (≤37)	1.57 (1.21–2.04)	1.54 (1.19–2.00)	1.50 (0.84–2.68)	1.48 (0.83–2.64)
**Size for Gestational age**				
<10%	1.33 (0.92–1.90)	1.28 (0.89–1.83)	1.91 (0.97–3.77)	1.90 (0.96–3.76)
10–90%	Referent	Referent	Referent	Referent
≥90%	0.53 (0.28–1.00)	0.55 (0.29–1.03)	0.38 (0.10–1.47)	0.39 (0.10–1.50)
**Apgar score-1 minute** ^4^				
<9	1.16 (0.81–1.65)	1.17 (0.82–1.68)	-----	-----

* Alpha-1-antitrypsin deficiency

^**†**^ Adjusted for maternal age and birth year;

^‡^ Adjusted for the imputed maternal smoking, maternal age, and birth year;

^1^ Collected for births 2003+;

^2^ Reference group: the 4th quartile;

^3^ Ponderal Index = birth weight (g)/ birth length (cm)³

^4^ Collected for 1991+

In sensitivity analyses, results unadjusted for imputed smoking status were similar when we adjusted for imputed smoking. Similarly, excluding mothers with a smoking history showed similar results to overall findings (Table 3, S2 and S3 Tables in S1 File). The risk of low birth weight babies also persisted when we restricted the analysis to term births (S4 Table in S1 File). Similarly, the risk of low birth weight (Risk Ratio (RR) = 1.77, 95% CI: 1.23–2.55), preterm birth (RR = 1.487, 95% CI: 1.14–1.96), shorter birth length (RR = 1.18, 95% CI: 1.05–1.32), and low ponderal index (RR = 1.08, 95% CI: 0.98–1.00), remained after excluding mothers with pre-eclampsia and premature rupture of membranes.

### Children with AATD and risk for adverse birth outcomes

The prevalence of AATD in the offspring of parents with AATD in our study was 37%. Our analysis showed a higher risk of children with AATD being low birth weight (RR = 1.64, 95% CI: 1.11–2.40) (S5 Table in S1 File). They were more likely to be in the lowest quartile of birth length (RR = 1.18, 95% CI: 1.04–1.33) and ponderal index (RR = 1.12–95% CI: 0.95–1.32). Also, when the child had AATD, their mothers were more likely to have a previous history of miscarriage (RR = 1.55, 95% CI: 1.02–2.37) when the mother was nonsmoking (S2 and S3 Tables in S1 File).

### Maternal AATD and risk for pregnancy and pre-pregnancy complications

Mothers with AATD had an increased prevalence of preeclampsia during pregnancy (Table 4) and had a higher risk of earlier diagnosis of pre-eclampsia, dyspnea, or COPD before the index pregnancy. These mothers had a higher risk of pre-eclampsia (RR = 2.64, 95% CI: 1.76–3.94), premature rupture of membranes (RR = 1.92, 95% CI: 0.92–4.00), and all alpha-1-related conditions (RR = 2.57, 95% CI: 1.84–3.59) during pregnancy. Similarly, there was a higher risk of COPD and all alpha-1-related conditions before pregnancy.

**Table 4:** Maternal AATD and the risk for pregnancy and pre-pregnancy complications and related conditions ^1^.

Complications	During pregnancy			Before pregnancy		
	Alpha1 Case N (%)	Non-cases N (%)	Crude RR	Alpha1 Case N (%)	Non-cases N (%)	Crude RR
Chronic obstructive pulmonary disease	<5 (0.3)	139 (0.0)	---	5 (1.6)	1426 (0.1)	23.12 (9.68–55.23)
Asthma ^2^	<5 (0.7)	3123 (0.2)	---	11 (3.6)	13875 (0.7)	5.23 (2.93–9.34)
Preeclampsia ^3^	22 (7.2)	54997 (2.7)	2.64 (1.76–3.94)	8 (4.04)	29246 (2.26)	2.14 (1.09–4.20)
Premature rupture of membranes	7 (2.3)	24009 (1.2)	1.92 (0.92–4.00)	<5 (0.3)	10151 (0.5)	---
All alpha-1-related conditions	31 (10.1)	79464 (3.9)	2.57 (1.84–3.59)	12 (3.9)	27277 (1.4)	2.90 (1.67–5.05)

^1^ Less than five mothers had a diagnosis of chronic bronchitis, hepatic cirrhosis, alcoholic cirrhosis, all-cause cirrhosis, prolonged oxygen dependency, dyspnea, emphysema, all-cause fatty liver disease, atopic dermatitis, rhinitis, panniculitis, pneumothorax, or vasculitis before or during the index pregnancy.

^2^ Asthma is not expected to be an alpha-1 complication but rather a misdiagnosis.

^3^ The "before pregnancy" preeclampsia diagnoses are the prevalence of preeclampsia among mothers with parity greater than one.

In analyzing congenital anomalies among children with AATD, only atresia of the bile duct (Q44.2) (RR = 455.79, 95% CI: 186.63–1113.17) had at least five diagnosed cases.

## Discussion

This is the first population-based epidemiologic study using a large cohort to determine the relationship between AATD and birth outcomes in mothers and children with this condition. Our study shows an association between AATD–when either the mother or child had AATD—and restricted fetal growth, whether defined as low birth weight, smallest quartile of birth length, low ponderal index, or small abdominal circumference. Furthermore, results were robust when we excluded factors that might have explained the results, including analyses restricted to nonsmoking mothers and when we excluded mothers with preterm birth, pre-eclampsia, or premature rupture of membranes. Hence, there appeared to be a robust association between AATD and IUGR.

Our findings support 2 case reports of a woman in her late 20s with AATD PiZZ phenotype whose pregnancy was complicated by fetal growth retardation [15], and a woman in her 40s with a history of pregnancies complicated by premature delivery, rupture of membranes, and miscarriages [13]. Although evidence suggests an increased risk of miscarriage among women with AATD [5, 6, 13], we only found an increased risk in mothers of children with AATD and not in the mothers themselves; however, miscarriage often goes unrecognized. If associations are confirmed, the adverse outcomes in babies born to mothers with AATD show the need for regular fetal monitoring to detect weight abnormalities.

AATD plays a role in numerous obstetric pathologies [11]. A previous study showed that AATD could be diagnosed from amniotic fluid and cord blood of unborn babies [16], and it also functions in the placenta. Although the role of AAT expression in the placenta is not fully known, AAT functions in the placenta as a substrate for the human serine protease high-temperature requirement A1 (HTRA)–a pro-inflammatory cytokine present to reduce inflammatory response and support trophoblast apoptosis [11, 17]. Therefore, a deficiency of AAT may promote an inflammatory response, affecting fetal growth.

Our study observed an increased risk of preterm birth in mothers with AATD. Our finding is consistent with a case report of repeated preterm birth in a woman with a confirmed diagnosis of AATD [13]. Preterm birth is a frequent cause of infant mortality globally, although technological advances in neonatology have improved survival rates. Previous investigations suggest early pregnancy-related inflammatory and immune events are part of the pathogenic mechanisms for preterm delivery. A proteomics study that analyzed biomarkers for preterm delivery in the first trimester of pregnancy suggested that low levels of AAT may be implicated [18]. Other similar proteomic studies on the amniotic fluid, vaginal fluid, and serum of women with PPROM and preterm birth indicated that low AAT levels were biomarkers for preterm birth [5, 19].

AAT also acts as an inhibitor of new vessel formation in small-diameter arteries. Thus, lower AAT levels in pregnant patients may impact the construction of small vessels in the uteroplacental circulation, causing decreases in uteroplacental blood flow and ultimately restricting blood circulation to the fetus [5]. Similarly, failure of the uterine vessels to develop may result in fetal growth restriction, which manifests as low birth weight or small for gestational age [20].

Likewise, the inhibitory activity of AAT is critical for preventing proteolytic tissue damage. Reduced levels of AAT have been reported in women with preeclampsia, which could explain the increased risk of preeclampsia in mothers with AATD in our study [15]. Also, low levels of AAT have been identified as a marker for preeclampsia, with the placenta contributing to the pathogenesis [21]. Therefore, impaired function of the uterine vessels could likely affect the placenta and contribute to increased pre-eclampsia.

Our study also observed a high placenta weight/ birth weight ratio. The placenta weight/birth weight ratio is a valuable indicator of the efficiency of the placenta, and a large ratio may indicate a nutrient supply deficit to the fetus. A proteomics study showed that low AAT levels are preeclampsia-related protein [22], and other studies also show that exogenous AAT administration may be protective against preeclampsia. AAT is cytoprotective and suppresses oxidative stress. Significantly low levels of AAT were found in the placenta of women with preeclampsia [23]. However, preeclampsia alone does not explain the association between AATD and pregnancy outcomes because excluding patients with preeclampsia did not change results. Therefore, a possible explanation for the high placenta/birth weight ratio may be the impairment of the apoptotic process mediated by AAT [11], but this requires further investigation at a molecular level. We didn’t find any association between AATD and size for gestational age and APGAR score; both are fetal well-being markers.

Our study found some associated maternal health conditions with AATD. For example, as other studies have seen, there was an increased risk of asthma, COPD, and other alpha-1-related conditions before or during pregnancy in mothers with AATD [24, 25]. However, the increased risk of asthma could be due to the misdiagnosis of early COPD [26]. Typically, AATD manifests in the pulmonary system with symptoms of COPD and emphysema [27]. Also, case reports of pregnant women with AATD reported deteriorating lung function tests (FEV1, FVC, and arterial blood gases) during the third trimester of pregnancy with worsening symptoms from the 37th week of gestation [3, 4]. Therefore, pregnant women with these symptoms should be screened for AATD.

Our study also showed an increased likelihood of delivery via Cesarean section in mothers with AATD, a finding consistent with multiple case reports [3, 4, 13]. Increased delivery via Cesarean section could be due to the development of complications in the late stages of pregnancy. However, excluding mothers with preeclampsia or PROM only slightly lowered point estimates. Cesarean sections are usually scheduled when the obstetrical conditions are unfavorable, or the fetal or maternal state does not make vaginal birth possible. Alpha-1-related conditions such as dyspnea that affect the mother’s respiration could be a reason for emergency Cesarean sections in these mothers, but our study design did not allow us to determine if this was the case. In Denmark, there has been a tendency for an increasing proportion of Cesarean sections over time, not necessarily due to complications. However, Cesarean remains low in Denmark compared to internationally [28].

Most AATD is not clinically recognized, with an estimated two to ten percent of AATD carriers ever diagnosed with the disease [29]. Given the estimated true prevalence, we calculated that in our study, 0.8–4% of pregnant mothers who presented with a history of asthma, 0.9–4.5% of mothers who presented with a history of dyspnea, and 3.5–17.5% of mothers with a history of COPD had AATD. If the subjects had a combination of asthma exacerbation and AATD, the risk of pregnancy-related complications could increase. These estimates suggest that AATD testing of pregnant mothers with these histories may be warranted, particularly in populations with common deleterious alleles.

Our study had several limitations. First, we expected an underdiagnosis of AATD. Our research shows that most people are not diagnosed with AATD until midlife. Hence, some of the mothers of the more recent births in our study have not sufficiently aged to have developed AATD symptoms and had the opportunity for diagnosis. However, AATD is rare, and this epidemiologic study is among the largest to examine its effect on birth outcomes. Also, we ascertained the diagnosis of AATD from ICD coding. There were no records of the genotype of AATD in our data nor information on the AAT concentration, and the pregnancy-related complications and adverse birth outcomes that we observed may differ by genotype and AAT level. We have no reason to believe that socioeconomic status is a confounder of this genetic disease, although we did not include it because of missing data. Another limitation of our study is that the first part of the study period (1973–1994) included only inpatient diagnoses; hence, some could have been missed. However, the remaining part of the study had both inpatient and outpatient diagnoses of AATD, and results were similar across the study periods. Furthermore, there were no records of augmentation therapy use in our database because it was not available as a general treatment strategy for AATD in Denmark during the years covered by our study [30]. We also could not adjust for passive smoking as that information was not in our data, but we adjusted for active smoking in mothers.

Strengths include that this population-based study is among the most extensive studies on pregnancy-related complications and birth outcomes in people with AATD, using prospectively collected medical records, e.g., without reliance on recall. This study was further strengthened by using objective records, including a reliable nationwide medical birth registry that captured cases over an extended period in a country with free access to healthcare for all residents.

In conclusion, AATD appears to be an underappreciated cause of fetal growth restriction and preterm birth. Since most people with AATD are asymptomatic during their childbearing years, diagnosis is rare. However, based on our findings, routine antenatal screening for AATD may need to be considered, particularly for mothers with a history of lung diseases. It is not known whether treatment with augmentation therapy might improve birth outcomes, and this may be considered in a future randomized clinical trial. Monitoring fetal growth and careful clinical follow-up in mothers with confirmed AATD should be a priority to minimize possible pregnancy-related complications.

## Supporting information

S1 File (PDF)

## Abbreviations

AAT: Alpha-1 antitrypsin
AATD: Alpha-1 antitrypsin deficiency
COPD: Chronic Obstructive Pulmonary Disease
ICD: International classification of disease
IUGR: Intrauterine growth restriction
PPROM: Preterm premature rupture of membrane
PROM: Premature rupture of membrane
