# Supplementary material for: Alpha-1 antitrypsin deficiency and pregnancy complications and birth outcomes: A population-based cohort study in Denmark
Source: PLoS One. 2024 Jan 2;19(1):e0296434. doi: 10.1371/journal.pone.0296434 (PMC10760838; doi:10.1371/journal.pone.0296434)
Supplement: S1 File — (PDF) [file pone.0296434.s001.pdf]

Supplemental Table 1: ICD Codes of Pregnancy Complications and Birth Outcomes

**Before and during pregnancy complications**	Chronic Bronchitis	Hepatic Cirrhosis	All-cause cirrhosis	Prolonged oxygen dependency	Chronic obstructive Pulmonary Disease	Dyspnea	Emphysema	All-cause Fatty liver disease	Asthma
**ICD- 8**	49100<br>49101<br>49102<br>49103<br>49104<br>49108<br>49109	571<br>57109<br>57110<br>57111<br>57119<br>57190<br>57191<br>57192<br>57193<br>571930<br>57194<br>57199	571<br>57109<br>57110<br>57111<br>57119<br>57190<br>57191<br>57192<br>57193<br>571930<br>57194<br>57199		490<br>49000<br>49001<br>49009<br>49100<br>49101<br>49102<br>49103<br>49104<br>49108<br>49109<br>49200<br>49201<br>49208<br>49209		49200<br>49201<br>49208<br>49209		493<br>49300<br>493000<br>49301<br>49302<br>49308<br>49309
**ICD- 10**	DJ41<br>DJ410<br>DJ411<br>DJ418	DK703<br>DK703A<br>DK744<br>DK745<br>DK746<br>DK746A<br>DK746B<br>DK746C<br>DK746D<br>DK746E<br>DK746G<br>DK746H	DK702<br>DK703<br>DK703A<br>DK704<br>DK704A<br>DK704B<br>DK704C<br>DK704D<br>DK740<br>DK740B<br>DK741<br>DK742<br>DK746<br>DK746A<br>DK746B<br>DK746C<br>DK746D<br>DK746E<br>DK746G<br>DK746H<br>DK766<br>DK766A<br>DK766B<br>DI850<br>DI859<br>DK717<br>DK717B<br>DK721<br>DK729<br>DK767	DZ998<br>DZ998A<br>DZ998B	DJ41<br>DJ410<br>DJ411<br>DJ418<br>DJ42<br>DJ429<br>DJ429A<br>DJ429B<br>DJ43<br>DJ430<br>DJ430A<br>DJ431<br>DJ431A<br>DJ432<br>DJ438<br>DJ439<br>DJ439A<br>DJ44<br>DJ440<br>DJ441<br>DJ448<br>DJ448A<br>DJ448B<br>DJ448C<br>DJ449	DR06<br>DR060<br>DR060A<br>DR061<br>DR062<br>DR063<br>DR064<br>DR065<br>DR065A<br>DR066<br>DR067<br>DR068<br>DR068A<br>DR068C	DJ43<br>DJ430<br>DJ430A<br>DJ431<br>DJ431A<br>DJ432<br>DJ438<br>DJ439<br>DJ439A	DK700<br>DK701<br>DK709<br>DK760<br>DK760A<br>DK760B<br>DK760C<br>DK758<br>DK759<br>DK759A	DJ45<br>DJ450<br>DJ450A<br>DJ451<br>DJ458<br>DJ459<br>DJ459A<br>DJ458<br>DJ469<br>DJ46

Supplemental Table 1 continued: ICD Codes of Pregnancy Complications and Birth Outcomes

**Before and during pregnancy complications**	Atopic dermatitis	Atopic dermatitis	Rhinitis	Panniculitis	Pneumo thorax	Pre-eclampsia	Premature rupture of membrane	Vasculitis
**ICD- 8**	69100<br>69101<br>69108<br>69109<br>69200<br>69201<br>69202<br>69208<br>69209<br>69210<br>69211<br>69212<br>69213<br>69219<br>69220<br>69221<br>69222<br>69223<br>69229<br>69230<br>69231<br>69232<br>69233<br>69239<br>69240	69241<br>69242<br>69243<br>69249<br>69259<br>69260<br>69261<br>69262<br>69263<br>69269<br>69279<br>69280<br>69281<br>69282<br>69283<br>69284<br>69285<br>69286<br>69287<br>69288<br>69289<br>69290<br>69291<br>69292<br>69299	50700<br>50701<br>50702<br>50703<br>50708<br>50709	68690<br>68692<br>68693<br>68694<br>68695<br>68699	51299	63700<br>63702<br>63703<br>63704<br>63709<br>63719<br>63799		76914<br>76919
**ICD- 10**	DL20<br>DL200<br>DL200A<br>DL208<br>DL208A<br>DL208B<br>DL208C<br>DL208D<br>DL209<br>DL22<br>DL229<br>DL229A<br>DL229B<br>DL229C<br>DL30<br>DL300<br>DL301<br>DL301A<br>DL301B<br>DL301C<br>DL302<br>DL302A<br>DL303<br>DL304<br>DL305	DL305A<br>DL308<br>DL308A<br>DL308B<br>DL308D<br>DL308F<br>DL308G<br>DL308H<br>DL308I<br>DL309<br>DH011<br>DH011A<br>DH011B<br>DH011C<br>DH011E<br>DH605<br>DH605A<br>DH605B<br>DH605C<br>DH605F<br>DH605H	DJ30<br>DJ300<br>DJ300A<br>DJ300B<br>DJ301<br>DJ301A<br>DJ301B<br>DJ301C<br>DJ302<br>DJ303<br>DJ303A<br>DJ304	DM793<br>DM793A<br>DM793B<br>DM793E<br>DM540<br>DM540A<br>DM540B	DJ93<br>DJ930<br>DJ931<br>DJ938<br>DJ939	DO11<br>DO119<br>DO119A<br>DO199B<br>DO119C<br>DO12<br>DO120<br>DO121<br>DO122<br>DO13<br>DO139<br>DO14<br>DO140<br>DO141<br>DO142<br>DO149	DO42<br>DO420<br>DO421<br>DO422<br>DO423<br>DO424<br>DO425<br>DO429	DL95<br>DL950<br>DL950A<br>DL951<br>DL958<br>DL958A<br>DL958B<br>DL959

Supplemental Table 2. Percentage distribution of maternal and child health of AATD *cases and non-cases (nonsmokers)

	Non-cases	Cases:	
		Mother	Children
	N= 833900	N= 78	N= 65
Maternal Health			
**Number of previous live births**			
0	555697 (66.6)	49 (62.8)	47 (72.3)
1	212777 (25.5)	25 (32.1)	16 (24.6)
>1	65426 (7.9)	4 (5.1)	<5
**Risky behavior in pregnancy** [1]	29357 (14.2)	<5	<5
**Maternal history of miscarriage**	142870 (17.1)	12 (15.4)	16 (24.6)
**Maternal history of Stillbirth**	1903 (0.8)	<5	<5
**Mother's height (cm)** [2] Mean (SD)	168.4 (6.4)	166.4 (7.4)	
**Mother's weight (kg)** [2] Mean (SD)	68.8 (14.6)	69.6 (16.1)	
**Maternal BMI** [2] Mean (SD)	24.2 (4.9)	25.1 (5.2)	
**Overweight (BMI≥25)** [2]	108851 (32.3)	12 (37.5)	
Child Health			
**Birthweight (g)** Mean (SD)	3545.6 (583.3)	3326.0 (678.3)	3384.2 (530.2)
<2500	33716 (4.0)	11 (14.1)	<5
2500-4000	640572 (76.8)	59 (75.6)	56 (86.2)
>4000	159612 (19.1)	8 (10.3)	7 (10.8)
**Placenta weight (g)** Mean (SD)	686.1 (171.1)	671.3 (135.6)	686.7 (158.2)
<570	141247 (23.3)	13 (21.0)	11 (23.9)
570-650	152044 (25.0)	15 (24.2)	9 (19.6)
660-760	155827 (25.7)	22 (35.5)	14 (30.4)
>760	158301 (26.1)	12 (19.4)	12 (26.1)
**Placental weight/birth weight ratio** Mean (SD)	0.2 (0.1)	0.2 (0.1)	0.2 (0.1)
1st quartile	160073 (26.4)	15 (24.2)	8 (17.4)
2nd quartile	154957 (25.5)	14 (22.6)	13(28.3)
3rd quartile	149848 (24.7)	11 (17.7)	11 (23.9)
4th quartile	142541 (23.5)	22 (35.5)	14 (30.4)
**Birth Length (cm)** Mean (SD)	52.0 (2.7)	51.2 (3.2)	51.6 (2.7)
<51	194939 (23.6)	24 (30.7)	17 (26.6)
51	115515 (14.0)	12 (15.4)	15 (23.4)
52-53	288133 (34.8)	29 (37.2)	17 (26.6)
>53	228699 (27.6)	13 (16.7)	15 (23.4)
**Head Circumference at birth (cm)** Mean (SD)	35.1 (1.8)	34.9 (2.1)	35.2 (1.8)
<34	91779 (15.0)	10 (16.7)	6 (13.0)
34-35	262018 (42.9)	27 (45.0)	22 (47.8)
36	137920 (22.6)	12 (20.0)	8 (17.4)
>36	119689 (19.6)	11 (18.3)	10 (21.7)
**Abdominal Circumference at birth (cm)** Mean (SD)	33.3 (2.4)	32.5 (2.6)	33.1 (2.3)
<32	110670 (18.6)	17 (29.3)	12 (26.7)
32-33	187972 (31.7)	22 (37.9)	14 (31.1)
34	114259 (19.2)	7 (12.1)	8 (17.8)
>34	180956 (30.5)	12 (20.7)	11 (24.4)
**Ponderal Index** [3] Mean (SD)	2.5 (0.4)	2.4 (0.2)	2.4 (0.2)
1st quartile	135609 (16.4)	18 (23.1)	13 (20.3)
2nd quartile	209126 (25.3)	21 (26.9)	17 (26.6)
3rd quartile	231155 (27.9)	22 (28.2)	23 (35.9)
4th quartile	251396 (30.4)	17 (21.8)	11 (17.2)
**Plural births**	15640 (1.9)	<5	<5
Cesarean section	31287 (14.5)	8 (42.1)	<5

* Alpha-1-antitrypsin deficiency;
[1] Collected for 1991-1996
[2] Collected for births 2003+;
[3] Ponderal Index = birth weight (g)/ birth length (cm)³;
Less than 1% were missing

Supplemental Table 3. Multivariable analysis of Children's AATD* and Risk for Adverse Birth Outcomes when the mother is non-smoking.

	Children with AATD (N=65) RR (95% CI)	
	**Crude**	**Adjusted †**
Maternal Health		
**Maternal history of miscarriage**		
Yes	1.44 (0.94-2.20)	1.55 (1.02-2.37)
**Birthweight (g)**		
<2500	-----	-----
2500-4000	Referent	Referent
>4000	0.56 (0.28-1.12)	0.57 (0.29-1.15)
**Placenta weight (g; quartiles)**		
<570	1.14 (0.77-1.70)	1.14 (0.77-1.69)
570-650	Referent	Referent
660-760	1.20 (0.87-1.67)	1.20 (0.87-1.67)
>760	1.12 (0.77-1.62)	1.14 (0.78-1.65)
**Placental weight/birth weight ratio (all births)**		
1st quartile	0.82 (0.48-1.38)	0.83 (0.49-1.41)
2nd quartile	1.07 (0.74-1.54)	1.07 (0.74-1.55)
3rd quartile	Referent	Referent
4th quartile	1.15 (0.81-1.63)	1.14 (0.81-1.62)
**Birth Length (cm; quartiles) [1]**		
<51(lowest quartile)	1.15 (0.83-1.60)	1.10 (0.79-1.53)
**Head Circumference at birth (cm; quartiles) [1]**		
<34 (lowest quartile)	0.86 (0.46-1.63)	0.84 (0.45-1.58)
**Abdominal Circumference at birth (cm; quartiles) [1]**		
<32 (lowest quartile)	1.37 (0.93-2.03)	1.37 (0.93-2.01)
**Ponderal Index [2]**		
1st quartile	1.34 (0.99-1.80)	1.33 (0.98-1.80)
**Plural births**		
Yes	-----	-----
**Cesarean section**		
Yes	1.63 (0.69-3.83)	1.49 (0.53-4.17)
**Gestational age (weeks)**		
Preterm birth (≤37)	-----	-----
**Size for Gestational age**		
<10 %	-----	-----
10-90 %	Referent	Referent
≥90 %	-----	-----
**Apgar score-1 minute [3]**		
<9	-----	-----

* Alpha-1-antitrypsin deficiency;
† Adjusted for mother's AATD-maternal age-and birth year;
[1] Reference group: the 4th quartile;
[2] Ponderal Index = birth weight (g)/ birth length (cm)^3; Reference group: the 4th quartile;
[3] Collected for 1991+;

Supplemental Table 4. Multivariable analysis of AATD* and Risk for Adverse Birth Outcomes among children born at term (37+ weeks)

	**Mother with AATD (N=226)**	
	**RR (95% CI)**	
	**Crude**	**Adjusted †**
Maternal Health		
**Number of Previous Live Births**		
0	Referent	Referent
1	0.88 (0.70-1.11)	0.92 (0.73-1.16)
>1	0.87 (0.55-1.37)	0.85 (0.56-1.29)
**Maternal history of miscarriage:**	0.81 (0.58-1.14)	0.85 (0.61-1.18)
**Maternal History of Stillbirth:**	3.37 (1.28-8.86)	3.03 (1.15-7.97)
**Mother's height (cm)** [1]		
<164	1.00 (0.68-1.48	1.01 (0.68-1.49)
164-168	Referent	Referent
169-172	0.31 (0.09-1.13)	0.31 (0.09-1.11)
≥173	0.93 (0.59-1.46)	0.92 (0.59-1.45)
**Mother's Weight (kg)** [1]		
<59	1.01 (0.61-1.67)	1.00 (0.60-1.66)
59-65	Referent	Referent
66-74	1.11 (0.75-1.65)	1.11 (0.75-1.65)
≥75	0.83 (0.45-1.54)	0.84 (0.45-1.58)
**Overweight (BMI≥25)** [1]: Yes	0.97 (0.60-1.58)	0.98 (0.60-1.60)
Child Health		
**Birthweight (g)**		
<2500	2.43 (1.49-3.96)	2.43 (1.49-3.97)
2500-4000	Referent	Referent
>4000	0.56 (0.37-0.85)	0.62 (0.41-0.94)
**Placenta weight (g)**		
<570	0.86 (0.53-1.39)	0.86 (0.53-1.39)
570-650	Referent	Referent
660-760	1.28 (1.03-1.59)	1.28 (1.03-1.59)
>760	1.05 (0.76-1.45)	1.05 (0.76-1.45)
**Placental weight/birth weight ratio (all births)**		
1st quartile	1.14 (0.82-1.57)	1.14 (0.83-1.57)
2nd quartile	1.17 (0.86-1.58)	1.17 (0.86-1.59)
3rd quartile	Referent	Referent
4th quartile	1.42 (1.14-1.78)	1.41 (1.12-1.76)
**Birth Length (cm; quartiles)** [2]		
<51 (lowest quartile)	1.23 (1.07-1.43)	1.22 (1.05-1.41)
**Head Circumference at birth (cm; quartiles)** [2]		
<34 (lowest quartile)	1.00 (0.71-1.41)	0.98 (0.61-1.60)
**Abdominal Circumference at birth (cm; quartiles)** [2]		
<32 (lowest quartile)	1.10 (0.71-1.71)	1.08 (0.69-1.71)
**Ponderal Index** [3,4]		
1st quartile	1.15 (0.98-1.36)	1.11 (0.95-1.31)
**Plural birth**	0.81 (0.20-3.23)	0.87 (0.22-3.47)
**Cesarean section**	1.77 (1.15-2.73)	1.82 (1.19-2.76)
**Size for Gestational age**		
<10 %	1.46 (0.97-2.20)	1.41 (0.94-2.12)
10-90 %	Referent	Referent
≥90 %	0.56 (0.30-1.05)	0.57 (0.30-1.08)
**Apgar score-1 minute** [4]		
<9	1.07 (0.71-1.62)	1.09 (0.72-1.64)

* Alpha-1-antitrypsin deficiency;
† Adjusted for the imputed maternal smoking, maternal age, and birth year;
‡ Adjusted for the imputed maternal smoking, paternal age, and birth year;
§ Adjusted for imputed maternal smoking, mother’s AATD, maternal age, and birth year;
[1] Collected for births 2003+;
[2] Reference group: the 4th quartile;
[3] Ponderal Index = birth weight (g)/ birth length (cm)^3;
[4] Collected for 1991+

Supplemental Table 5. Multivariable analysis of Children's AATD* and Risk for Adverse Birth Outcomes.

	Children with AATD (all) (N=254)	
	RR (95% CI)	
	Crude	Adjusted †
Maternal Health		
**Maternal history of miscarriage**		
Yes	0.96 (0.70-1.30)	1.06 (0.78-1.43)
**Birthweight (g)**		
<2500	1.67 (1.13-2.46)	1.64 (1.11-2.40)
2500-4000	Referent	Referent
>4000	0.65 (0.44-0.98)	0.73 (0.49-1.10)
**Placenta weight (g; quartiles)**		
<570	1.15 (0.81-1.65)	1.16 (0.81-1.66)
570-650	Referent	Referent
660-760	1.22 (0.90-1.66)	1.22 (0.90-1.65)
>760	1.25 (0.94-1.67)	1.25 (0.93-1.67)
**Placental weight/birth weight ratio (all births)**		
1st quartile	0.96 (0.62-1.47)	0.96 (0.62-1.47)
2nd quartile	1.07 (0.75-1.53)	1.07 (0.75-1.53)
3rd quartile	Referent	Referent
4th quartile	1.22 (0.93-1.62)	1.23 (0.93-1.62)
**Birth Length (cm; quartiles)** [2]		
<51	1.22 (1.08-1.38)	1.18 (1.04-1.33)
**Head Circumference at birth (cm; quartiles)** [2]		
<34 (lowest quartile)	0.82 (0.46-1.47)	0.84 (0.47-1.52)
**Abdominal Circumference at birth (cm; quartiles)** [2]		
<32 (lowest quartile)	1.31 (0.90-1.89)	1.41 (0.98-2.04)
**Ponderal Index** [3]		
1st quartile	1.16 (0.99-1.37)	1.12 (0.95-1.32)
**Plural births**		
Yes	0.58 (0.15-2.31)	0.69 (0.18-2.72)
**Cesarean section**		
Yes	0.68 (0.33-1.40)	0.72 (0.35-1.48)
**Gestational age (weeks)**		
Preterm birth (≤37)	1.21 (0.87-1.69)	1.20 (0.86-1.66)
**Size for Gestational age**		
<10 %	1.44 (0.96-2.17)	1.38 (0.92-2.08)
10-90 %	Referent	Referent
≥90 %	0.97 (0.57-1.66)	1.01 (0.59-1.73)
**Apgar score-1 minute** [4]		
<9	1.18 (0.81-1.73)	1.18 (0.81-1.73)

* Alpha-1-antitrypsin deficiency;
† Adjusted for imputed maternal smoking, mother's AATD, maternal age, and birth year;
[1] Collected for births 2003+;
[2] Reference group: the 4th quartile;
[3] Ponderal Index = birth weight (g)/ birth length (cm)^3; Reference group: the 4th quartile;
[4] Collected for 1991+;
